# Short-term results of ab-interno trabeculotomy using Kahook Dual Blade in patients with primary congenital glaucoma

**DOI:** 10.5935/0004-2749.20210048

**Published:** 2021

**Authors:** Larissa Fouad Ibrahim, Senice Alvarenga Rodrigues Silva, Tiago Santos Prata, Fabio Nishimura Kanadani

**Affiliations:** 1 Department of Ophthalmology, Instituto de Olhos Ciências Médicas, Belo Horizonte, MG, Brazil; 2 Department of Ophthalmology, Universidade Federal de São Paulo, São Paulo, SP, Brazil; 3 Department of Ophthalmology, Mayo Clinic, Jacksonville, Florida, USA

**Keywords:** Primary congenital glaucoma, Kahook Dual Blade, Ab-interno trabeculotomy, Trabecular meshwork, Glaucoma incisional surgery, Glaucoma congênito primário, Kahook Dual Blade, Trabeculotomia ab-interno, Malha trabecular, Cirurgia incisional de glaucoma

## Abstract

The aim of this study is to present the results of ab-interno trabeculotomy using Kahook Dual Blade in patients with primary congenital glaucoma. An ab-interno trabeculotomy using a dual blade device was performed in three eyes of two patients with the diagnosis of primary congenital glaucoma. One of them in the left eye and the other patient in both eyes. In the first patient, an adequate response was achieved after the intraocular pressure reduced from 36 mmHg to 14 mmHg. The second patient did not respond adequately to the procedure, and high intraocular pressure levels persisted in both eyes after the procedure. The indication of Kahook Dual Blade ab-interno trabeculotomy in primary congenital glaucoma must be cautious and more studies are needed to establish its efficacy and the best indications. Seems that this procedure should not be indicated for primary congenital glaucoma treatment.

## Abstract (pt)

O objetivo deste estudo é apresentar os resultados da trabeculotomia ab-interno com Kahook Dual Blade em pacientes com glaucoma congênito primário. Foi realizada trabeculotomia ab-interno com dispositivo de lâmina dupla em 3 olhos de 2 pacientes com diagnóstico de glaucoma congênito primário. Um deles no olho esquerdo e o outro paciente nos dois olhos. No primeiro paciente, houve resposta adequada uma vez que a pressão intraocular diminuiu de 36 mmHg para 14mmHg. O segundo paciente não respondeu adequadamente ao procedimento, mantendo altos níveis de pressão intraocular em ambos os olhos após o procedimento. A indicação da trabeculotomia ab-interno com o Kahook Dual Blade no glaucoma congênito primário deve ser cautelosa e são necessários mais estudos para estabelecer a eficácia e as melhores indicações. Parece que esse procedimento não deve ser indicado no tratamento do glaucoma congênito primário.

## INTRODUCTION

Primary congenital glaucoma (PCG) is characterized by elevation of intraocular pressure (IOP) in the first year of life as a result of reduced drainage of aqueous humor by the trabecular meshwork (TM). It occurs sporadically in about 1 in 10,000 births^(1-4)^, resulting in blindness in approximately 10% of cases^(1,2)^.

Pharmacological therapy is generally not efficient in PCG. Goniotomy is usually the procedure of choice if corneal transparency allows performing it. Conventional trabeculotomy is indicated in presence of corneal opacities. In refractory cases, surgical alternatives such as trabeculectomy, glaucoma drainage implants, and cyclodestructive procedures could be considered^(2)^.

The Kahook Dual Blade^®^ (KDB; New World Medical, Rancho Cucamonga, CA, USA) is an ophthalmic surgical blade designed to remove the TM membrane more completely than traditional goniotomy with less collateral damage^(5)^. To the best of our knowledge, we report here the first three cases of patients with PCG in Brazil who underwent the ab-interno trabeculotomy procedure.

## CASE REPORTS

### Case 1

A 7-month-old male infant was referred with PCG diagnosis. His mother reported cesarean delivery, with a gestational age of 39 weeks, birth weight of 4300g, gestational diabetes mellitus, photophobia, and tearing in both eyes (OU), more evident in the left eye (OS). The presence of mild corneal edema and buphthalmos in OS was observed at biomicroscopy. The right eye (OD) showed no alterations. The IOP was 16 mmHg in the OD and 36 mmHg in the OS (Perkins applanation tonometer = PKT). In this eye, brinzolamide 1% and brimonidine 0.2% were initiated. With the patient under sedation, examinations revealed the following in the OD and the OS, respectively: a horizontal corneal diameter of 10 and 13 mm, axial length of 21.32 and 24.7 mm, IOP of 08 and 28 mmHg (PKT) and 04 and 23 mmHg (iCare^®^), central corneal thickness of 550 and 720 µm, and normal disc in the OD and a cup-to-disc ratio of 0.6 in the OS, with an evident asymmetry. KDB ab-interno trabeculotomy was performed with good angle visualization, without intercurrences. Four months after, the patient had an IOP of 10 mmHg in the OD and 14 mmHg in the OS (PKT) under sedation, without any ocular hypotensive medication.

### Case 2

A 7-month-old female infant was referred with suspected PCG. The mother reported cesarean delivery, with a gestational age of 38 weeks and birth weight of 4250g. Ophthalmological evaluation revealed in OU: mild corneal edema without leukoma, with a bidigital hypertensive touch and IOP of 24 mmHg (iCare^®^). Examinations under sedation identified the presence of Haab’s striae, an IOP of 28 mmHg (PKT), central corneal thickness of 580 µm and fundoscopy showing extensive and deep excavations in OU. Ab-interno trabeculotomy was performed in OU without intercurrences. Six months later, the patient’s IOP was 24 mmHg in OU (iCare^®^), even using Latanoprost. A conventional trabeculotomy was performed in OU, without intercurrence. Improvement in corneal edema was noted postoperatively, and the patient’s IOP was 14 mmHg in the OD and 28 mmHg in the OS (iCare^®^). A second conventional trabeculotomy was performed in the OS. Three months later, the IOP was 12 and 16 mmHg (PKT) in the OD and OS, respectively, without medications.

The following procedure was used for the KDB ab-interno trabeculotomy technique. Under general anesthesia, a 1.2-mm × 1.4-mm side port blade was used to create a temporal wound. Acetylcholine chloride 1:100 was injected into the anterior chamber to constrict the pupil and help visualize the angle structures. Cohesive ophthalmic viscoelastic was injected into the anterior chamber. The patient’s head was rotated until good nasal iridocorneal angle visualization was achieved with direct gonioscopy lens. The KDB was introduced through the wound into the nasal angle. Then it was used to remove the nasal TM for about four clock hours. It was noted that the TM pealed and fell away in this area.

## DISCUSSION

Ab-interno trabeculotomy with KDB is already indicated for the treatment of open angle glaucoma and ocular hypertension^(5)^. However, its use is poorly evaluated in patients with PCG. There is one case report in the literature describing the use of the KDB for PCG treatment in OU of a 13-month-old patient. After three months, while receiving hypotensive eye drops bilaterally, the patient’s IOP was reduced from 43 to 21 mmHg in the OD after a single procedure and from 44 to 34 mmHg in the OS after three surgeries, requiring glaucoma drainage implant placement. The author claimed that the asymmetry in the level of maldevelopment possibly resulted in the poor response to surgery in the left eye^(6)^. Although our first case showed buphthalmos and an increased axial length, the surgical response was satisfactory. In the second case, both eyes demonstrated poor surgical response even presenting mild ocular maldevelopments.

Khouri, AS and colleagues published another case report in which treatment using KDB was successful in a child with glaucoma secondary to the extraction of bilateral congenital cataract. In this case, the eye was left aphakic^(6,7)^. In our small case series, KDB was used successfully in the first patient. In the case of the second patient, the IOP remained elevated in OU, and further surgical reintervention was necessary. IOP reduction occurred only after conventional trabeculotomy. We still cannot determine a predictive factor that would suggest a better response to ab-interno trabeculotomy.

**Figure 1:**
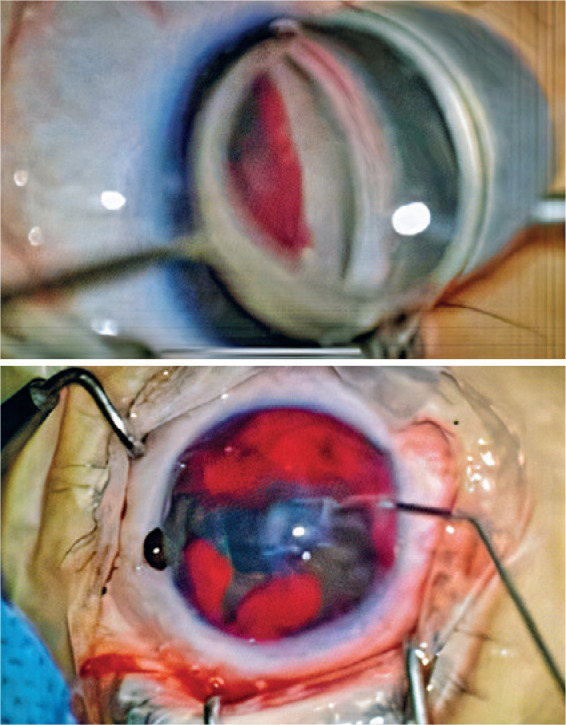
Expressive bleeding after TM membrane removal.

One reason for the low success rate (one-third of PGC eyes) of KDB might be the degree of maturation of the conventional aqueous humor outflow pathway. Regarding the surgery, some technical issues have to be considered, including a shallow anterior chamber in phakic eyes, expressive bleeding during the removal of the TM membrane (Figure 1), and the risk of iris-lens diaphragm anteriorization after intracameral of acetylcholine chloride injection.

KDB could be a less traumatic surgical option in PCG, facilitating future surgical reassessments. It is also believed that there is a reduced risk of future aqueous humor obstruction of Schlemm’s channel^(6)^.

We still do not feel confident indicating KDB ab-interno for PCG treatment. Additional short-and long-term studies are needed to establish the efficacy and indications for ab-interno trabeculotomy using KDB in patients with PCG.

## References

[1] Shields MB, Allingham RR, Damji KF (2008). Shields tratado de glaucoma.

[2] Brink DB, Brasil MB, Brink GB (2015). Perfil epidemiológico dos pacientes com glaucoma
congênito atendidos no Hospital Regional de São
José. Rev Bras Oftalmol.

[3] Paolera MD. (2008). Avaliação do gene CYP1B1 em pacientes com glaucoma
congênito primário.

[4] Dias JF, Almeida HG, Prata Junior AP. (2010). Glaucoma.

[5] Young CC, Seibold LK, Aref AA. (2018). Kahook Dual Blade ab interno trabeculectomy.

[6] Harvey MM, Schmitz JW. (2020). Use of ab interno Kahook Dual Blade trabeculectomy for treatment
of primary congenital glaucoma. Eur J Ophthalmol.

[7] Khouri AS, Wong SH. (2017). Ab interno trabeculectomy with a dual blade: surgical technique
for childhood glaucoma. J Glaucoma.

[8] Biglan AW. (2006). Glaucoma in children: Are we making progress?. J AAPOS.

